# Supplementary material for: TBKIN: Threshold-based explicit selection for enhanced cross-modal semantic alignments
Source: PLoS One. 2025 Jun 10;20(6):e0325543. doi: 10.1371/journal.pone.0325543 (PMC12151420; doi:10.1371/journal.pone.0325543)
Supplement: Attachment — Submitted filename: Response to Reviewers.docx [file pone.0325543.s001.docx]

**Manuscript ID:** PONE-D-24-60420

**Paper Title:** Threshold-based knowledge integration vision-language model

Dear Editor,

We sincerely appreciate your letter and the insightful comments provided by the reviewers on our manuscript titled *Threshold-based knowledge integration vision-language model* (PONE-D-24-60420). The reviewers’ feedback is invaluable, offering both constructive guidance for refining and enhancing our paper and significant insights that will inform our ongoing research. We have meticulously examined each comment and implemented the necessary revisions to address them comprehensively. To uphold transparency and streamline the revision review process, we have systematically annotated all modifications within the manuscript. Deleted content is meticulously marked with strikethroughs, while newly incorporated material addressing journal requirements and editorial feedback is distinctly highlighted in **red**. Revisions in response to **Reviewer #1**’s comments are marked in **blue**, and those addressing **Reviewer #2**’s comments are clearly identified in **orange**. This structured approach ensures clarity and facilitates a comprehensive assessment of the revisions made in accordance with the feedback provided.

We extend our heartfelt gratitude to the editor and reviewers for their meticulous and constructive feedback, which has profoundly strengthened the rigor and clarity of our manuscript. Their insightful suggestions have notably improved the theoretical framework, experimental methodology, and the overall reliability of our findings. We are deeply appreciative of the opportunity to resubmit the revised manuscript and eagerly await further guidance to ensure its excellence.

Once again, we sincerely thank you for your invaluable expertise, patience, and support throughout this process.

Best regards,

Zihan Guo, Xiang Shen, Chongqing Chen

## Response to journal requirements

Journal requirements:

When submitting your revision, we need you to address these additional requirements.

**1. Please ensure that your manuscript meets PLOS ONE’s style requirements, including those for file naming.**

**Response:** We have meticulously reviewed our manuscript in accordance with the PLOS ONE style templates and implemented the necessary revisions. Specifically, we have:

1. Revised the formatting of tables (**Tables 1-5**) and adjusted the citation style for multiple tables (**line** **633**).
2. Renamed all figure files to align with the journal’s requirements.
3. Applied the “et al.” convention for references with more than six authors, listing only the first six authors followed by “et al.” (**references** **6, 12, 15, 18, 19, 22, 27, 33, 37, 38**).

**2. In the online submission form, you indicated that your data is available only on request from a third party. Please note that your Data Availability Statement is currently missing [the name of the third party contact or institution / contact details for the third party, such as an email address or a link to where data requests can be made]. Please update your statement with the missing information.**

**Response:** We sincerely apologize for the oversight in our initial description of data availability. It was incorrect to state that the data are available only upon request from a third party. In fact, our data are openly accessible and have been deposited in public repositories. We will promptly update the statement to reflect this information accurately and ensure full compliance with journal requirements.

**3.** **Please note that your Data Availability Statement is currently missing [the repository name and/or the DOI/accession number of each dataset OR a direct link to access each database]. If your manuscript is accepted for publication, you will be asked to provide these details on a very short timeline. We therefore suggest that you provide this information now, though we will not hold up the peer review process if you are unable.**

**Response:** Thank you for your reminder. We have now updated our **Data Availability Statement** as follows:

All relevant datasets are publicly accessible from the VQA database (<https://visualqa.org/index.html>) and the REC database (<https://cocodataset.org/#home>).

We confirm that these links provide direct access to the datasets used in our study.

## Response to additional editor comments

Additional editor comments:

**Among the comments from the reviewers, the authors are expected to particularly clarify the novelty and the contribution of this work, and clearly differentiate the proposed approach from existing models.**

**Response:** We sincerely appreciate your feedback and the opportunity to further clarify the novelty and contribution of our work. In response to the reviewers’ comments, we have carefully elaborated on the unique aspects of our proposed approach and how it distinguishes itself from existing models. We have addressed this in detail in our responses to **Reviewer #1**’s comments **4**, **5**, and **7** and **Reviewer #2**’s comment **1**. Specifically, the revisions include, but are not limited to, the following key aspects:

1. Highlighting the innovative aspects of our methodology and its theoretical advancements.
2. Clearly differentiating our approach from prior models by emphasizing its unique features and performance improvements.
3. Providing a more detailed discussion of the practical contributions of our work to the field.

We hope these clarifications adequately address your concerns and demonstrate the distinct novelty and contribution of our study. Should any additional modifications be required, we are happy to address them promptly.

## Response to reviewers’ comments

Reviewers’ comments:

Reviewer’s responses to questions

Comments to the author

**1. Is the manuscript technically sound, and do the data support the conclusions?**

The manuscript must describe a technically sound piece of scientific research with data that supports the conclusions. Experiments must have been conducted rigorously, with appropriate controls, replication, and sample sizes. The conclusions must be drawn appropriately based on the data presented.

**Reviewer #1: Yes**

**Reviewer #2: Partly**

**Response:** We sincerely appreciate the opportunity to address the concerns regarding the technical soundness and data support for the conclusions in our manuscript. In response to the comments from reviewers, we have carefully revised the manuscript to ensure its rigor, clarity, and validity. Specifically, the revisions include, but are not limited to, the following key aspects:

1. **Clarity and intent of the paper:** We have improved the overall clarity of the manuscript by refining the language, structure, and presentation to ensure that the research objectives, methodology, and contributions are well-articulated and easy to follow.
2. **Initialization method and learning strategy of the TBKIN model:** We have provided a more detailed and transparent explanation of the initialization process and learning strategy employed for the proposed TBKIN model to enhance reproducibility and technical rigor.
3. **Improved presentation of figures and tables:** We have revised the figures and tables to ensure they are more intuitive, clearly labeled, and effectively support the conclusions drawn in the manuscript.

These revisions directly address the concerns raised by the reviewers and strengthen the technical soundness of the manuscript. For detailed responses, please refer to our replies to **Reviewer #1’s comment 4** and **Reviewer #2’s comments 1-7**. We believe the revised manuscript now provides robust data and analysis that fully support the conclusions drawn. Should any additional modifications be required, we are happy to address them promptly.

**2. Has the statistical analysis been performed appropriately and rigorously?**

**Reviewer #1: I Don’t Know**

**Reviewer #2: No**

**Response:** We deeply appreciate the opportunity to address the concern regarding the appropriateness and rigor of the statistical analysis in our manuscript. In response to the comments from reviewers, we have carefully revised the manuscript to ensure the statistical analysis is appropriately executed and comprehensively described. The revisions include, but are not limited to, the following key aspects:

1. **Model details and learning strategy:** We have elaborated on the specific details of the proposed model, including its learning strategy and the use of the VLM model, to enhance the clarity and reproducibility of our approach.
2. **Clearer description and analysis of experiments and results:** We have improved the description of the model’s initialization method, presented experimental results in a more graphical and intuitive manner, and provided a detailed comparison with state-of-the-art models. These revisions ensure a robust and transparent analysis of our findings.

For more detailed responses, please refer to our replies to **Reviewer #1’s comment 7** and **Reviewer #2’s comments 2-6**. We believe these revisions have strengthened the statistical rigor and overall quality of the manuscript. Should any additional modifications be required, we are happy to address them promptly.

**3. Have the authors made all data underlying the findings in their manuscript fully available?**

The PLOS Data policy requires authors to make all data underlying the findings described in their manuscript fully available without restriction, with rare exception (please refer to the Data Availability Statement in the manuscript PDF file). The data should be provided as part of the manuscript or its supporting information, or deposited to a public repository. For example, in addition to summary statistics, the data points behind means, medians and variance measures should be available. If there are restrictions on publicly sharing data—e.g. participant privacy or use of data from a third party—those must be specified.

**Reviewer #1: Yes**

**Reviewer #2: No**

**Response:** Thank you for highlighting the importance of data availability in our manuscript. We greatly appreciate the opportunity to address this concern and ensure full compliance with the PLOS Data policy. In our study, we utilized the VQA 2.0 dataset and three REC datasets derived from COCO images, all of which are publicly available and stored in well-established repositories. To address **Reviewer #2**’s concern and further enhance transparency, we have updated the **Data Availability Statement** to explicitly detail how these datasets can be accessed without restriction. Specifically, the relevant data are openly available through the following sources:

**VQA 2.0 Dataset:** Accessible at <https://visualqa.org/index.html>.

**REC Datasets (COCO-based):** Accessible at <https://cocodataset.org/#home>.

We sincerely apologize for any oversight in our initial submission regarding the clarity of data accessibility. We are committed to upholding the highest standards of data sharing and transparency in our research. We believe these revisions fully address the concerns raised by **Reviewer #2** and align our manuscript with the requirements of the PLOS Data policy. Should any additional modifications be required, we are happy to address them promptly.

**4.** **Is the manuscript presented in an intelligible fashion and written in standard English?**

PLOS ONE does not copyedit accepted manuscripts, so the language in submitted articles must be clear, correct, and unambiguous. Any typographical or grammatical errors should be corrected at revision, so please note any specific errors here.

**Reviewer #1: Yes**

**Reviewer #2: No**

**Response:** We sincerely thank the reviewers and the editor for their valuable feedback regarding the presentation and language of our manuscript. In response to the comments from reviewers, we have thoroughly revised the manuscript to ensure it is presented in an intelligible fashion and written in clear, standard English. The revisions include, but are not limited to, the following key improvements:

1. **Enhanced title:** The manuscript title has been revised to be more comprehensive and reflective of the study’s scope.
2. **Structured and complete abstract:** The **Abstract** has been restructured to provide a clearer and more complete summary of the research.
3. **Enriched introduction:** The **Introduction** section has been expanded to provide a more thorough background and context for the study.
4. **Clearer research motivation:** The motivation behind the research has been articulated more explicitly to better highlight its significance.
5. **Explanation of advantages over existing methods:** We have provided a more detailed explanation of how our approach outperforms existing methods.
6. **Improved clarity and readability:** The overall clarity and readability of the manuscript have been enhanced to ensure it is accessible to a broad audience.

For detailed responses to the specific comments, please refer to our replies to **Reviewer #1**’s comments **1-4**, **7**, and **8**, as well as **Reviewer #2**’s comments **1-7**. We believe these revisions have significantly improved the presentation and language quality of the manuscript. Should any additional modifications be required, we are happy to address them promptly.

**5. Review comments to the author**

**Reviewer #1:**

**1. The title of this paper is not informative enough, please revised.**

**Response:** We sincerely thank you for your valuable feedback regarding the title of our paper. In response to your observation that the title was not sufficiently informative, we have carefully revised it to better emphasize the core contributions and focus of our work. The updated title is now:

**“****TBKIN: Threshold-based explicit selection for enhanced cross-modal semantic alignments”**

We believe this version provides clearer and more precise insights into the key contributions and scope of our research. Should any additional modifications be required, we are happy to address them promptly.

**2.** **Abstract is not well written (IMRaD is a standard format for writing abstracts in an orderly manner .... Such as i. Introduction, ii Methodology, iii Results and iv Discussions). A summary of the result is missing from this abstract. Please rewrite the abstract.**

**Response:** We sincerely thank you for your valuable feedback regarding the **Abstract**. In response to your observation that the **Abstract** was not well-structured and lacked a summary of results, we have thoroughly revised it in alignment with the IMRaD format. The updated **Abstract** is provided below for your review:

We believe the revised **Abstract** now offers a more structured, informative, and complete overview of our research. Should any additional modifications be required, we are happy to address them promptly.

**3. The introduction can be enriched by briefly describe the state-of-the-art in the title of this study and provide more recently related references to support foundation of this studies to better context for the current research. I will recommend citing recently published related papers.**

**Response:** We sincerely thank you for your valuable feedback and insightful suggestions. In response to your recommendation, we have enriched the **Introduction** by providing a concise description of a recent advancement in the field related to our research (**lines** **71-74**). Additionally, we have incorporated your suggested references, as well as another relevant recent publication, to strengthen the foundation of our work. These additions provide a comprehensive background and highlight how our study aligns with current state-of-the-art developments in the field. Should any additional modifications be required, we are happy to address them promptly.

**4.** **The problem that author is trying to address in this paper is not clear yet, (clearer motivation for study is required).**

**Response:** We sincerely thank you for your valuable feedback. We acknowledge that the motivation for our study was not sufficiently clear in the original manuscript. To address this, we have thoroughly revised the **Introduction** to explicitly state the problem we aim to solve and the significance of our research. Specifically:

1. **Clear problem statement:** We added a concise description of the research problem in the **Introduction**, emphasizing its relevance and the gaps in existing approaches (**lines** **83-102**).
2. **Enhanced motivation:** We expanded the discussion on the motivation for our study, highlighting its potential impact and practical applications (**lines** **102-139**).
3. **Alignment with objectives:** We ensured that the problem statement and motivation are directly aligned with the objectives and contributions outlined in the manuscript.

These revisions provide a clearer rationale for our study and better position its importance within the broader research context. We believe these changes address your concern and strengthen the overall clarity and focus of the manuscript. Should any additional modifications be required, we are happy to address them promptly.

**5. In 4-5- lines, authors should summarize the literature gaps identified before starting the methodology (Kindly relate your work in the context of existing work, what are the shortcomings of existing studies?)**

**Response:** We sincerely thank you for your insightful feedback and constructive suggestion regarding the need to summarize the literature gaps prior to introducing the methodology. We fully agree that situating our work within the context of existing studies and explicitly addressing their limitations is essential to highlight the novelty and significance of our research. In response to your comment, we have carefully revised the **Introduction** section of the manuscript. Specifically, we have incorporated a dedicated paragraph (**lines** **83-104**) that systematically outlines the key shortcomings of existing studies relevant to our research. By identifying these gaps, we have established a more compelling rationale for our methodology and emphasized how our work addresses these unmet needs in the field (**lines** **105-139**).

We believe this revision not only strengthens the manuscript’s coherence but also provides a clearer foundation for understanding the contributions of our study. Should any additional modifications be required, we are happy to address them promptly.

**6. Author should re-check the correctness of equations especially equations 3 to 8.**

**Response:** We sincerely thank you for your careful attention to the equations in our manuscript, particularly **equations 3 to 8**. We fully acknowledge the critical importance of ensuring the accuracy and correctness of all mathematical formulations in our work. In response to your comment, we meticulously re-examined all equations in the manuscript, with a specific focus on **equations 3 to 8**. Through a thorough review and verification process, we confirm that these equations are mathematically correct and precisely represent the relationships described in the text.

We greatly appreciate your diligence in highlighting this aspect of our manuscript. While no errors were identified, your comment prompted us to conduct an additional rigorous review, further enhancing the reliability and robustness of our work. Should any additional modifications be required, we are happy to address them promptly.

**7. Can you further explain how your work succeed beyond REF 5, 6, and 15?**

**Response:** We sincerely thank you for your valuable comment regarding the need to further clarify how our work succeeds beyond the approaches proposed in **REF 6**, **7**, and **18** (previously **REF 5**, **6**, and **15**). We have carefully addressed this concern in the revised manuscript by explicitly comparing TBKIN with these models and highlighting our key innovations (**lines** **601-616**). Below, we provide a detailed explanation of the limitations of these reference models and the unique contributions of our approach:

While the models in **REF 6**, **7**, and **18** represent state-of-the-art advancements in vision-language modeling—achieving significant progress in pre-training strategies and cross-modal alignments—they share a critical limitation: the inability to explicitly filter out irrelevant information during downstream tasks. This oversight often leads to suboptimal performance in noisy or complex scenarios, where the ability to discern and disregard distracting data is essential for ensuring both accuracy and robustness. As a result, the persistent interference of irrelevant information hampers their effectiveness in real-world applications, particularly those involving cluttered visuals and intricate textual inputs, thereby limiting their overall utility. In contrast, our proposed TBKIN introduces a novel threshold-based knowledge integration network that addresses this limitation by mitigating the influence of irrelevant information. By employing a sparse selection method during fine-tuning, TBKIN effectively filters out distracting data, ensuring robust performance even in highly noisy environments. This innovation is a significant departure from the approaches in **REF 6**, **7**, and **18**, which lack explicit mechanisms for handling irrelevant information.

Once again, we extend our sincere gratitude for your constructive feedback, which has significantly strengthened our manuscript and provided a clearer demonstration of the novelty and effectiveness of our proposed framework. We believe these revisions will enhance the readers’ understanding of how TBKIN sets itself apart from existing approaches in the field. Should any additional modifications be required, we are happy to address them promptly.

**8. Services of language expert are required (be mindful of tenses).**

**Response:** We sincerely thank you for your valuable feedback regarding language quality. We have engaged a professional language editing expert to meticulously revise the manuscript, ensuring proper verb tense usage, improved sentence structure, and adherence to standard academic English. To enhance the clarity and ease of reviewing the revisions in response to your other comments, we have highlighted the retouched manuscript content in **red** for your convenience.

We believe these revisions have significantly enhanced the clarity and readability of the manuscript. Should any additional modifications be required, we are happy to address them promptly.

**9.** **Image quality needs to be improved.**

**Response:** We sincerely thank you for your valuable feedback regarding image quality. In response, we have carefully enhanced the resolution and clarity of our figures according to the journal’s guidelines. The revised figures have been uploaded to **PACE** and have successfully passed the required quality checks.

We believe these improvements meet the journal’s standards for visual presentation. Should any additional modifications be required, we are happy to address them promptly.

**10. Ensure that all Figures, Tables and Equations are properly labeled and referenced.**

**Response:** We sincerely thank you for your valuable feedback regarding the labeling and referencing of figures, tables, and equations. In response, we have meticulously reviewed and revised all elements to ensure proper labeling, formatting, and accurate referencing in accordance with the journal’s guidelines. Specifically, we have updated the table formats (**Tables 1-5**) and adjusted the citation styles for multiple tables (**line** **633**) to enhance clarity and consistency.

We believe these changes now meet the journal’s standards. Should any additional modifications be required, we are happy to address them promptly.

**Reviewer #2:**

**1.** **The clarity of the paper should be improved. It often is hard to understand, and the text leaves it unclear, what the intentions are.**

**Response:** We sincerely thank you for your constructive feedback regarding the clarity of the paper. In response, we have made comprehensive revisions to improve the overall clarity and coherence of the manuscript, particularly addressing the concerns raised by you and incorporating **Reviewer #1**’s comments **4**, **5**, and **7**. Specifically, we have:

1. Clearly articulated the problem we aim to solve and the significance of our research in the **Introduction** section (**lines** **83-139**).
2. Explicitly compared TBKIN with existing models in the **Experiments and results** section, emphasizing our key innovations and contributions (**lines** **601-616**).

We believe these revisions have significantly enhanced the clarity and readability of the manuscript, ensuring that the intentions and contributions of our research are now more clearly conveyed. Should any further improvements be needed, we are happy to address them promptly.

**2. The introduction leaves it unclear TBKIN learning strategy on intra-modal and cross-modal knowledge based on unified scene graphs and masking strategies.**

**Response:** We sincerely thank you for your valuable comment regarding the clarity of TBKIN’s learning strategy on intra-modal and cross-modal knowledge based on unified scene graphs and masking strategies. Based on this feedback, we have revised the corresponding section in the **Introduction** (**lines 107-128**) to provide a more detailed and coherent explanation of the methodology. Specifically, we have:

1. Clarified the process of modeling intra-modal knowledge and cross-modal knowledge.
2. Explained how to extract the fusion representation of image and text.
3. Detailed the multi-task learning objective and fine-tuning strategy.

We believe these revisions have addressed your concern and improved the clarity and comprehensiveness of the **Introduction**. Should any further improvements be needed, we are happy to address them promptly.

**3. Please clarify: Is the VLM only used during training or during rollout? For the method without VLM, in which stages has it been left out?**

**Response:** We sincerely thank you for your valuable questions regarding the usage of our VLM. The VLM is employed in both the training and rollout stages. However, there are key differences in its application across these stages:

1. During training, the primary objective of the VLM is to complete the knowledge masking tasks (based on three distinct masking strategies) and the image-text matching task, which are designed to enhance cross-modal semantic alignments (**lines** **425-436**). During rollout, the VLM is utilized to accomplish downstream tasks, specifically visual question answering and referring expression comprehension (**lines** **450-454**).
2. We do not use the fine-tuning strategy based on threshold selection during training to allow the model to learn more comprehensive intra-modal and cross-modal semantic alignments (**lines** **497-499**). We do use this strategy during rollout to filter out irrelevant and noisy information from the input data, thereby further optimizing the model’s performance on downstream tasks (**lines** **450-454**).

We hope this clarification addresses your concerns. Should any further improvements be needed, we are happy to address them promptly.

**4. Please explain initialization, as this is barely mentioned in the rest of the main text.**

**Response:** We sincerely thank you for your valuable feedback regarding the explanation of the initialization process. We acknowledge that a more detailed explanation of initialization is essential for clarity and reproducibility. Below, we provide a comprehensive clarification:

1. **Initialization details:** TBKIN’s parameters are initialized using the publicly available **BERT-base model**. This initialization ensures that the model starts from a robust semantic representation, which is crucial for effective downstream performance (**lines** **527-529**).
2. **Training configuration:** Following initialization, the model is trained for **up to 40 epochs** with a **batch size of 512** to optimize its performance. For detailed parameter settings related to the **structural knowledge masking strategy**, we refer readers to **reference 18** in our manuscript (**lines** **530-534**).

We hope this explanation adequately addresses your concerns. Should any further improvements be needed, we are happy to address them promptly.

**5. Ablations regarding the VQA and REC policy are not clear. In general, the architecture of the policy is not clear from the paper.**

**Response:** We sincerely thank you for your insightful feedback regarding the clarity of the ablation experiments and the architecture of the policy. We have carefully addressed these concerns and revised the manuscript to improve the explanation of both aspects. Below, we provide a detailed response to your comment.

1. **Clarity of the ablation experiments:** To enhance the clarity of our ablation experiments, we have restructured and refined the description of the **Ablation studies** section (**lines 630-648**). The revised text now explicitly outlines the purpose of the ablation study, the role of the threshold-based selection strategy, and the comparison with another sparse attention method. These revisions aim to ensure that the methodology, motivation, and findings of the ablation experiments are presented in a clear and accessible manner.
2. **Architecture of the policy:** We have significantly expanded and refined the description of the policy architecture in the manuscript. As detailed in our responses to your **first four comments** and **Reviewer #1’s comments (particularly comments 2, 4, and 7)**, we have provided a more comprehensive explanation of key components.

We believe these revisions address your concerns and improve the clarity and comprehensibility of the manuscript. Should any further improvements be needed, we are happy to address them promptly.

**6. Could you provide some graphical visualization of the table? I believe this would make the claimed more apparent.**

**Response:** We sincerely thank you for your valuable suggestion regarding the graphical visualization of the tables. We agree that visual representations can significantly enhance the clarity and interpretability of the results, making the claims more apparent and accessible to readers. In response to this feedback, we have added a concise line graph (**Fig 5**) to visualize the results in **Table 2**. This graphs clearly illustrates the impact of different threshold values on the performance of TBKIN for the VQA task.

To avoid redundancy and ensure the manuscript remains focused, we have chosen not to add graphical visualizations for the other tables. Instead, we have ensured that the results in **Tables 3-5** are presented in a clear and structured format, with detailed discussions to guide the reader through the key findings.

We believe that the addition of the line graph for **Table 2**, combined with the clarity of the other tables, effectively addresses your suggestion and enhances the overall impact of the manuscript. Should any further improvements be needed, we are happy to address them promptly.

**7. The figs are hard to understand.**

**Response:** We sincerely thank you for your valuable feedback regarding the clarity of the figures. We fully acknowledge that the figures were initially difficult to interpret, and we deeply apologize for any inconvenience caused. In response to this feedback, we have added detailed legends for **Fig 1**, **3**, **5**, and **6** to ensure better readability. In the main text, we have included detailed descriptions of the figures, ensuring that each element is thoroughly contextualized and easier to follow (**lines 263-272, 277-282, 325-333, 480-497, and 662-680**).

These revisions have significantly improved the clarity of the figures, making them more intuitive and accessible to readers. We believe these changes address your concerns and enhance the overall quality of the manuscript. Should any further improvements be needed, we are happy to address them promptly.
